# Efficient Transmission and Characterization of Creutzfeldt–Jakob Disease Strains in Bank Voles

**DOI:** 10.1371/journal.ppat.0020012

**Published:** 2006-02-24

**Authors:** Romolo Nonno, Michele A. Di Bari, Franco Cardone, Gabriele Vaccari, Paola Fazzi, Giacomo Dell'Omo, Claudia Cartoni, Loredana Ingrosso, Aileen Boyle, Roberta Galeno, Marco Sbriccoli, Hans-Peter Lipp, Moira Bruce, Maurizio Pocchiari, Umberto Agrimi

**Affiliations:** 1 Department of Food Safety and Veterinary Public Health, Istituto Superiore di Sanità, Viale Regina Elena, Rome, Italy; 2 Department of Cell Biology and Neurosciences, Istituto Superiore di Sanità, Viale Regina Elena, Rome, Italy; 3 Division of Neuroanatomy and Behaviour, Institute of Anatomy and Centre for Neuroscience, University of Zürich, Zürich, Switzerland; 4 Neuropathogenesis Unit, Institute for Animal Health, Edinburgh, United Kingdom; University of Toronto, Canada

## Abstract

Transmission of prions between species is limited by the “species barrier,” which hampers a full characterization of human prion strains in the mouse model. We report that the efficiency of primary transmission of prions from Creutzfeldt–Jakob disease patients to a wild rodent species, the bank vole *(Clethrionomys glareolus),* is comparable to that reported in transgenic mice carrying human prion protein, in spite of a low prion protein–sequence homology between man and vole. Voles infected with sporadic and genetic Creutzfeldt–Jakob disease isolates show strain-specific patterns of spongiform degeneration and pathological prion protein–deposition, and accumulate protease-resistant prion protein with biochemical properties similar to the human counterpart. Adaptation of genetic Creutzfeldt–Jakob disease isolates to voles shows little or no evidence of a transmission barrier, in contrast to the striking barriers observed during transmission of mouse, hamster, and sheep prions to voles. Our results imply that in voles there is no clear relationship between the degree of homology of the prion protein of the donor and recipient species and susceptibility, consistent with the view that the prion strain gives a major contribution to the species barrier. The vole is therefore a valuable model to study human prion diversity and, being susceptible to a range of animal prions, represents a unique tool for comparing isolates from different species.

## Synopsis

Prions are unconventional infectious agents that cause fatal neurodegenerative diseases. The transmission of prions between species is considered a rare event because it is limited by the “species barrier.” Nevertheless, in the past 10 y, more than 180 people worldwide died with variant Creutzfeldt–Jakob disease (vCJD) following consumption of bovine spongiform encephalopathy (BSE)–contaminated food. The vCJD crisis highlights the need for experimental approaches that are able to characterize human prions and to estimate the risk of animal prions for man. The authors used a new animal model, the bank vole, which appears to address these issues. They observed that these rodents are highly susceptible to sporadic Creutzfeldt–Jakob disease (sCJD) and genetic Creutzfeldt–Jakob disease (gCJD), as well as to several animal prions. Transmission to voles indicates that sCJD is caused by at least two distinct prion strains. Surprisingly, voles challenged with gCJD isolates do not show a species barrier, while prions from closely related rodent species encounter a clear barrier in transmitting to voles. Inoculation of voles with scrapie-related and BSE-related strains from several species suggests that the prion strain, and not the donor species, is the major determinant of prion transmissibility between different species. The authors conclude that the vole model is a valuable tool for comparing animal and human prions.

## Introduction

Transmissible spongiform encephalopathies (TSEs) are a group of fatal neurodegenerative diseases of humans and animals, caused by unconventional infectious agents known as prions. They are characterized by the accumulation of a disease-associated isoform (PrP^Sc^) of the host-encoded cellular prion protein (PrP^C^). According to the protein-only hypothesis, prions are composed mainly or exclusively of PrP^Sc^. Although apparently devoid of any nucleic acid, prions exist as different infectious strains with characteristic pathogenetic properties [1], which can be characterized from their different disease phenotypes in an inbred animal host. The prion hypothesis equates strains to different self-propagating conformational variants of PrP^Sc^ [2], which parallel the diversity of physicochemical properties of PrP^Sc^ observed in human and animal prion diseases [3–6]. The electrophoretic mobility and the relative level of glycosylation of the protease-resistant fragment of PrP^Sc^ are the basis for the molecular classification of TSEs.

Sporadic Creutzfeldt–Jakob disease (sCJD) represents the most common human TSE [7], occurring worldwide with an incidence of about 1.7 cases per million people per year [8], and has an apparently spontaneous origin. Genetic Creutzfeldt–Jakob disease (gCJD) [9] is associated with mutations of the prion protein–gene and accounts for about 10% of Creutzfeldt–Jakob disease (CJD) cases [8]. Other genetic TSEs are Gerstmann–Sträussler–Scheinker disease (GSS) and fatal familial insomnia. The emergence of variant Creutzfeldt–Jakob disease (vCJD) [10], a new disease linked to bovine spongiform encephalopathy (BSE) [11,12], highlights the zoonotic potential of TSEs.

Prion diversity is revealed by transmission to laboratory animals, but this approach can be seriously limited by the “species barrier” effect, which hampers a full characterization of human prion strains in the mouse model [11,13]. Animal models that are suitable for studies with sCJD or gCJD therefore represent a major advance in understanding the extent to which various clinico-pathological forms represent different strains, and whether atypical forms are caused by novel prion strains.

Although early studies with transgenic mice suggested that the degree of PrP sequence homology is responsible for the species-barrier effect [14], a number of studies since then, in addition to the results reported here, have challenged this view [11,15,16]. The main strategy for overcoming the species barrier from human to mice has been the generation of transgenic mouse lines over-expressing human PrP or chimeric mouse–human PrP [2,17–22]. Such mice usually propagate human prions more efficiently than wild-type mice, although with variable results depending on the prion strain. For example, vCJD transmits more readily to wild-type mice than to the humanized transgenic models. Recently, the term “transmission barrier” rather than “species barrier” has been proposed to account for the variable ability of prion strains to cross barriers between species [23]. To improve on the traditional mouse-based model and to deepen our understanding of the species barrier, we carried out transmission studies of human prions to the bank vole *(Clethrionomys glareolus),* a wild rodent species which has proved to be susceptible to prions from a range of sources [24] (U. Agrimi et al., unpublished data).

## Results

### Voles Are Susceptible to Human Prions

In sCJD, the PrP genotype at position 129, codifying for methionine (M) or valine (V), and the electrophoretic mobility of the protease-resistant unglycosylated fragment of PrP^Sc^ (21 kDa for type 1 and 19 kDa for type 2) are correlated with disease phenotype and are the basis for the classification of sCJD into six distinct subtypes: MM1/MV1, VV2, MV2, MM2, MM2-thalamic, and VV1 [25]. The efficiency of transmission of sCJD to voles and mice varied according to different subtypes. Voles were highly susceptible to MM1 and MV1 sCJD, with all voles succumbing to disease after short survival times (Table 1). Voles inoculated with MM2 sCJD succumbed to disease with longer survival times (Table 1), while MV2 and VV2 sCJD did not induce disease up to 500 d after inoculation. The emerging picture is in agreement with recent findings in humanized transgenic models, showing a general tendency of MM1/MV1 sCJD to transmit with higher attack rates and shorter incubation times than type 2 cases [19,20,21]. Intriguingly, this phenomenon appears to parallel disease duration in human sCJD cases, in that type 2 cases are usually associated with longer survival times than type 1 cases [26].

**Table 1:**
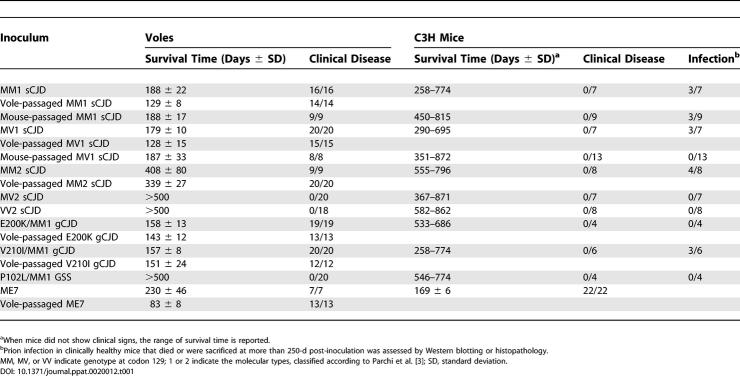
Survival Times of Voles and C3H Mice Infected with Human TSEs

Voles were also highly susceptible to prions from gCJD cases associated with the PrP mutations V210I and E200K (Table 1). By contrast, the P102L GSS case has not given rise to clinical disease up to 500 d after inoculation. In comparison with human TSEs, the prototype mouse-passaged scrapie strain ME7 transmitted with a mean survival time that was intermediate between type 1 and type 2 CJD cases (Table 1).

C3H mice were less susceptible than voles to all human cases inoculated (Table 1). Mice did not show obvious clinical signs and were sacrificed or died at ages typical for uninoculated mice. However, based on PrP^Sc^ accumulation assessed by Western blot and/or spongiform changes assessed by histopathology, 40%–50% of mice surviving for more than 250 d after inoculation with MM1/MV1 sCJD, MM2 sCJD, and V210I gCJD showed unequivocal signs of prion infection and were thus considered infected (unpublished data). These results are similar to those previously reported for primary transmissions of MM1 sCJD to other mouse strains [11]. By contrast, MV2 sCJD, VV2 sCJD, E200K gCJD, and GSS did not show evidence of transmission in any recipient mice.

### Some CJD Isolates Adapt to Voles with Little or No Evidence of Transmission Barrier

The transmission-barrier effect appears to consist of several different elements: a low efficiency of infection, the selection of agent strains that replicate more rapidly in the new species, and differences in pathogenesis between interspecies and intraspecies transmissions. The extent of a transmission barrier can be inferred by the decrease in survival time between the primary transmission and subsequent passage in the new host species. To investigate whether the ease of transmission of certain human TSEs to voles is the result of a low transmission barrier, we set up secondary transmissions. MM1 and MV1 sCJD gave similar mean survival times of ~130 d post-inoculation (Table 1), with a significant shortening of ~30% compared to primary transmission. In comparison, MM2 sCJD gave a long incubation period on the second passage as well as on the first passage (Table 1), with only a slight decrease compared with the first passage. Intriguingly, secondary transmission of E200K or V210I gCJD gave no significant decrease in survival time compared to the primary transmission (Table 1), suggesting that the efficiency of transmission of gCJD was not affected by a human–vole species barrier. On the other hand, the mouse-passaged scrapie strain ME7 encountered a striking transmission barrier and adapted to voles as a very fast strain (Table 1).

### PrP^Sc^ Produced in CJD-Infected Voles Has Similar Molecular Features to the Corresponding Human Inocula

**Figure 1:**
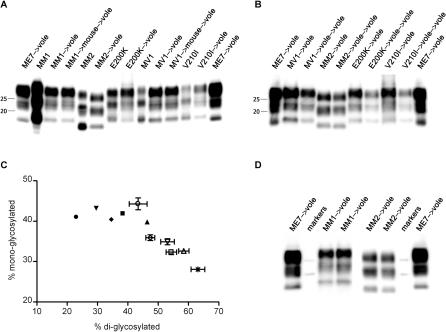
Determination of the Molecular Type of PrP^Sc^ Produced in Voles following Transmission of sCJD and gCJD (A) Immunoblot of proteinase K–resistant PrP^Sc^ from sCJD and gCJD subtypes in human patients and after first passage in voles. PrP^Sc^ produced in voles after primary transmission of mouse-passaged MM1 sCJD and MV1 sCJD, and of the mouse-adapted scrapie strain ME7 is also shown. The identity of the brain sample is designated above each lane. (B) Comparison of proteinase K–resistant PrP^Sc^ produced in voles following first and second passages of MV1 sCJD, MM2 sCJD, E200K gCJD, and V210I gCJD. PrP^Sc^ produced in voles after first passage of the mouse-adapted scrapie strain ME7 is also shown. The identity of the brain sample is designated above each lane. (C) Scatter-graph of proportions of di-glycosylated and mono-glycosylated PrP^Sc^ in human patients (denoted by filled circles, filled inverted triangles, filled diamonds, filled squares, and filled upright triangles) and voles (denoted by open circles, open inverted triangles, open diamonds, open squares, open upright triangles, and asterisks), with MM1 sCJD (denoted by filled squares and open squares), MV1 sCJD (denoted by filled diamonds and open diamonds), MM2 sCJD (denoted by filled circles and open circles), V210I gCJD (denoted by filled inverted triangles and open inverted triangles), E200K gCJD (denoted by filled upright triangles and open upright triangles), and ME7 (denoted by asterisks). (D) Comparison of proteinase K–resistant PrP^Sc^ produced in voles following inoculation with MM1 sCJD, MM2 sCJD, and the mouse-adapted scrapie strain ME7. Strep-tagged molecular markers (25 kDa and 20 kDa) are shown. The identity of the brain samples is designated above each lane.

All voles succumbing to prion diseases accumulated high levels of protease-resistant PrP^Sc^ in their brains. We studied the biochemical characteristics of human and vole PrP^Sc^ by comparing their electrophoretic mobility and glycoform pattern after protease digestion (Figure 1). Voles inoculated with type 1 human PrP^Sc^ (MM1 sCJD, MV1 sCJD, E200K gCJD, V210I gCJD) accumulated PrP^Sc^ with a protease-resistant unglycosylated fragment of the same size as the human counterpart (Figure 1A). In MM2 sCJD, the molecular weight of both human and vole unglycosylated PrP^Sc^ was of type 2, ~2 kDa lower than type 1 (Figure 1A). These PrP^Sc^ molecular patterns were unchanged after vole-to-vole transmission (Figure 1B). As the size of the protease-resistant unglycosylated fragment is believed to reflect the conformation of PrP^Sc^ [2], these findings show that the subtype-specific conformations of human PrP^Sc^ have been faithfully reproduced in voles. Interestingly, the molecular weight of this PrP^Sc^ fragment from voles inoculated with the mouse-adapted scrapie strain ME7 was intermediate between types 1 and 2 (Figure 1A, 1B, and 1D).

The relative amounts of the three major bands observed on immunoblot further characterizes subtype-specific PrP^Sc^ fragments [4,25,27]. The glycoform pattern of the human PrP^Sc^ was characterized by the expected dominance of the mono-glycosylated PrP^Sc^ fragment in sCJD and V210I gCJD, while E200K gCJD had similar levels of di-glycosylated and mono-glycosylated PrP^Sc^ fragments [27] (Figure 1A and 1C). Vole PrP^Sc^ was generally more glycosylated than human PrP^Sc^. MM2 sCJD–affected voles showed similar levels of di-glycosylated and mono-glycosylated PrP^Sc^ fragments, while di-glycosylated PrP^Sc^ was dominant in voles with MM1/MV1 sCJD and gCJD (Figure 1A and 1C). Similar changes in the glycosylation pattern associated with the same strain after transmission to mice have been reported [12,21]. The glycoform pattern of PrP^Sc^ in voles inoculated with ME7 was different from that obtained in voles inoculated with all human cases, being characterized by the highest proportion of di-glycosylated PrP^Sc^ (Figure 1C).

### Lesion Profiles and PrP^Sc^ Deposition in Voles Show Subtype-Specific Patterns

Spongiform neurodegeneration was the most prominent finding in voles affected with CJD, accompanied by gliosis and neuronal loss (Figure S1). These changes were localized in specific brain regions with little variability amongst individual voles. In voles with sCJD and gCJD, caudal brain regions were mainly spared.

**Figure 2:**
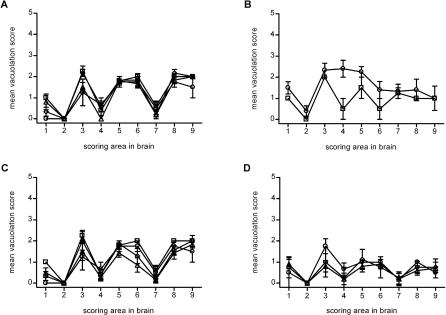
Lesion Profiles in Voles and Mice following Transmission of sCJD and gCJD Brain-scoring positions are medulla (1), cerebellum (2), superior colliculus (3), hypothalamus (4), thalamus (5), hippocampus (6), septum (7), retrosplenial and adjacent motor cortex (8), and cingulate and adjacent motor cortex (9). (A) Lesion profiles in voles infected with MM1 sCJD (denoted by open squares), MV1 sCJD (denoted by open circles), V210I gCJD (denoted by open diamonds), and E200K gCJD (denoted by open upright triangles). (B) Lesion profiles in voles infected with MM2 sCJD (denoted by open squares) and the mouse-passaged scrapie strain ME7 (denoted by open circles). (C) Lesion profiles in voles infected with human sCJD (denoted by open squares and open circles) and mouse-passaged sCJD (denoted by open diamonds and open upright triangles), MM1 sCJD (denoted by open squares and open diamonds), and MV1 sCJD (denoted by open circles and open upright triangles). (D) Lesion profiles in C3H mice infected with MM1 sCJD (denoted by open circles), MV1 sCJD (denoted by open upright triangles), and V210I gCJD (denoted by open diamonds).

To characterize the disease phenotype of affected voles, we analyzed the patterns of vacuolar degeneration in selected areas of the brain, as represented by the “lesion profile” (Figure 2). The lesion profile is a well-established semi-quantitative method for representing the targeting of vacuolation to different brain regions, and reliably discriminates between TSE strains in mice [11,28]. The MM1 sCJD, MV1 sCJD, V210I gCJD, and E200K gCJD cases produced identical patterns of vacuolar degeneration in voles (Figure 2A), characterized by grey-matter spongiform degeneration of the superior colliculi, thalamic nuclei, and hippocampus, and the retrosplenial and cingulate cortices. The medulla oblongata was less severely involved, while the cerebellum, hypothalamus, and septum showed only occasional vacuolation. Voles infected from the MM2 sCJD case (Figure 2B) displayed a pattern of vacuolation in the medulla, cerebellum, and thalamus similar to that observed with MM1/MV1 sCJD, but their lesion profile was characterized by a lower spongiform change in the hippocampus and cortices, while septal nuclei were more severely involved. Voles inoculated with mouse scrapie ME7 (Figure 2B) had a distinct lesion profile characterized by severe vacuolation of the hypothalamus, which was mainly spared by human prions.

Spongiform changes in positive mice with MM1 sCJD, MV1 sCJD, and V210I gCJD were generally mild, and this was consistent with the absence of clinical disease. The lesion profiles paralleled those observed in voles (Figure 2D) and are in agreement with previous reports of sCJD-infected mice [11,29].

**Figure 3:**
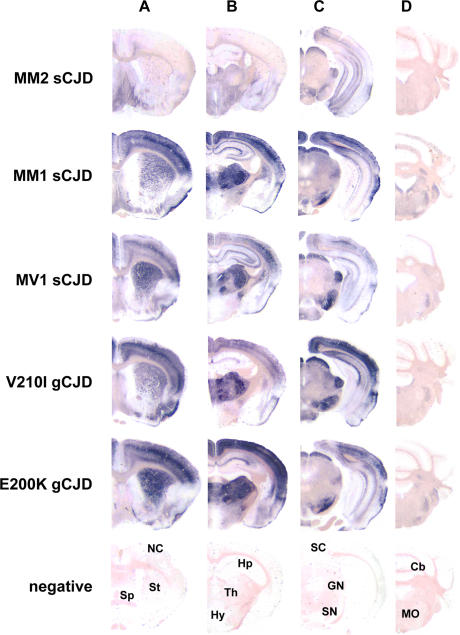
Regional Distribution of Protease-Resistant PrP^Sc^ in Voles following Transmission of sCJD and gCJD PET blots of coronal sections of the forebrain (telencephalon in [A] and diencephalon in [B]), midbrain (C), and hindbrain (D) in voles infected with MM2 sCJD, MM1 sCJD, MV1 sCJD, V210I gCJD, and E200K gCJD. At the lower part of the figure, the labeled coronal sections of a negative control brain are shown. NC, neocortex; Sp, septum; St, striatum; Hp, hippocampus; Th, thalamus; Hy, hypothalamus; SC, superior colliculus; GN, geniculate nuclei; SN, substantia nigra; Cb, cerebellum; MO, medulla oblongata.

Analysis of the distribution of PrP^Sc^ in the brain, assessed by paraffin-embedded tissue (PET) blot (Figure 3), further strengthened the similarity of type 1 sCJD and gCJD, which showed indistinguishable patterns of PrP^Sc^ accumulation, primarily in the cerebral cortex, striatum, thalamus, substantia nigra, hippocampus, and in the nuclei of the visual pathways. On the other hand, MM2 sCJD was characterized by a stronger PrP^Sc^ accumulation in the septum, lateral habenular nuclei, and hypothalamus, with only sparse labeling in the cerebral cortex and striatum. Immunohistochemistry for PrP^Sc^ showed mainly a punctate or synaptic staining in type 1 sCJD and in gCJD, while MM2 sCJD was characterized by a coarse, mostly intracellular, pattern and by frequent staining of the rim of vacuoles (Figure S1).

### C3H Mice Do Not Show Disease upon Serial Passage of sCJD, but Propagate Infectivity with Strain Properties Identical to the Original Human Inocula

During adaptation to a new host, prions can either maintain their original strain properties, as assessed by passage back into the original species, or can change in a manner consistent with the isolation of variant strains with shorter incubation periods in the new host [30]. In an attempt to adapt sCJD to C3H mice, we set up secondary transmission experiments. C3H mice infected with mouse-passaged MM1 sCJD and MV1 sCJD did not show clinical signs of prion disease and died or were sacrificed after long survival times (Table 1). Only three out of nine mice inoculated with mouse-passaged MV1 sCJD had low levels of PrP^Sc^ (unpublished data), while none of the mice inoculated with MM1 sCJD showed evidence of prion infection. However, in another study of MM1 sCJD in different mouse strains, it was possible to transmit infection from mouse to mouse, but the incubation periods remained extremely long (M. Bruce, unpublished data). Lack of adaptation after successful primary transmission was unexpected, although a similar phenomenon was recently reported [22] in transgenic mice expressing human PrP with V at codon 129 infected with BSE. Our findings suggest that C3H mice propagate sCJD prions at a level insufficient for sustaining the disease upon serial passage.

To address this hypothesis, we inoculated bank voles with the same mouse-passaged sCJD used for secondary transmissions in mice. Voles had very short survival times (188 and 187 d post-infection [dpi], respectively; see Table 1) and a 100% attack rate, showing that the brains of mice inoculated with sCJD contained high levels of infectivity, incompatible with persistence of the original human inoculum. Strikingly, the survival times, the biochemical characteristics of PrP^Sc^, and the brain-lesion profiles of voles inoculated with mouse-passaged sCJD were identical to those observed after direct transmission of the original sCJD isolates (Table 1; Figures 1A and 2C). From these experiments, we conclude that a subset of mice infected with type 1 sCJD propagated high levels of infectious prions which carried strain-specific information similar to that of the original human prions. Recently, several experimental approaches have emphasized the possible occurrence of a subclinical carrier-state in recipient animals, after either prion interspecies [31,32] or intraspecies [33,34] transmissions, and implications for public health have been emphasized [23,35]. Our data add further concern because they indicate that animals remaining clinically healthy after interspecies transmission may replicate prions that transmit inefficiently to individuals of the same species but are highly infectious for a third species.

### Transmission Barrier Depends on Prion Strain, rather than PrP Homology

**Figure 4:**
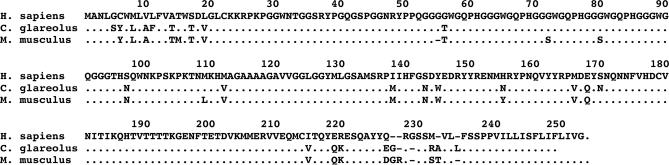
Alignment of Human, Vole, and Mouse Prion Protein–Amino Acid Sequences The sequence numbers of the human *(Homo sapiens)* amino acids are indicated and refer to the residue under the final digit. In the vole *(C. glareolus)* and the mouse *(Mus musculus)* sequences, identical residues to the human are indicated as dots.

Differences in amino acid sequence between donor PrP^Sc^ and recipient PrP^C^ have been proposed to be one of the main factors contributing to prion species barriers [14]. However, the reasons for the extraordinary sensitivity of voles to sCJD and gCJD, compared to the low susceptibility of mice, are not easily deduced from the comparison of PrP sequences (Figure 4). Indeed, the prion proteins of voles and mice display high homology and have several amino acid substitutions compared to human PrP. Taking into consideration the central portion of the PrP sequence, which has been proposed to play a major role in determining the species barrier [17], the vole and the mouse prion proteins differ at three positions, which are Met^109^Asn^154^Asn^169^ in the vole and Leu^108^Tyr^153^Ser^168^ in the mouse. The corresponding human positions are Met^109^His^154^Ser^169^, suggesting that homology at position 109 between the vole and the human PrP sequences might play a role in the ease of transmission observed with some human TSEs.

**Figure 5:**
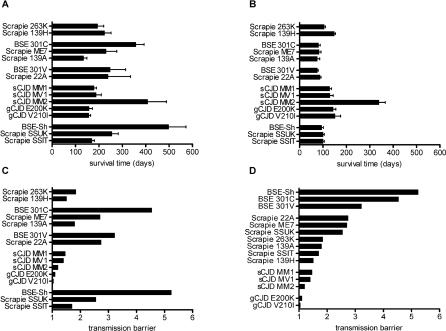
Comparison of Transmission Barriers of Human, Sheep, Mouse, and Hamster Prions following Transmission to Voles In (A–C), inocula are grouped according to the sequence homology of mature PrP between the donor species and voles, from the least to the most divergent (golden hamster, 2.9% divergence; C57BL mouse strain, 2.9% divergence; VM mouse strain, 3.4% divergence; human, 7.1% divergence; and sheep, 7.1% divergence). In (D), inocula are ranked according to the magnitude of the transmission barrier and are grouped according to the original prion source. (A) Survival times following first passage in voles of hamster-passaged scrapie strains 263K and 139H, C57BL mice-passaged BSE strain 301C, C57BL mice-passaged scrapie strains ME7 and 139A, VM mice-passaged BSE strain 301V, VM mice-passaged scrapie strain 22A, human sCJD (MM1, MV1, and MM2 subtypes) and gCJD (E200K and V210I subtypes), sheep-passaged BSE, and sheep natural scrapie isolates from the United Kingdom (SSUK) and Italy (SSIT); error bars represent standard deviation. (B) Survival times following second passage in voles of the same transmissions as in (A); error bars represent standard deviation. (C and D) The magnitude of each transmission barrier is calculated as the ratio of mean survival times observed following first and second passage (a value of 1 denotes an absence of transmission barrier).

It is worth noting that all animal models suitable for the study of sCJD and gCJD prions are Met at the corresponding human residue 109. Even single amino acid substitutions at this position of the prion protein can influence the efficiency of transmission of prions to mice [36,37] and voles [24]. To further evaluate the relative contributions of TSE agent strain and PrP sequence homology in determining the transmission barrier, we inoculated bank voles with scrapie-related and BSE-related strains from species with different PrP sequences, such as C57Bl mice, VM mice, hamsters, and sheep (Figure 5). While the short survival times observed overall in these transmission studies suggest that the high susceptibility of voles to TSEs is not restricted to human prions (Figure 5A), the decrease in survival time observed in secondary transmissions implies that scrapie-related and BSE-related strains, unlike gCJD, encounter a clear transmission barrier in voles (Figure 5B).

The extent of each transmission barrier was inferred by the reduction in survival times between primary and secondary transmissions (Figure 5C and 5D). The variable transmission barriers observed with different prion strains from the same donor species (Figure 5C) clearly point to the prion strain as a determinant of the outcome of interspecies transmission in bank voles, as opposed to PrP sequence homology with the donor species. Conversely, the magnitude of the transmission barrier varied according to the original source of the prion isolate, irrespective of the donor species (Figure 5D).

## Discussion

We studied the transmission characteristics of human prions in a new animal model, the bank vole, which showed an unforeseen susceptibility to sCJD and gCJD. Some subtypes of sCJD and gCJD gave very short survival times and faithful propagation of their molecular phenotype in voles. These findings are unprecedented for wild-type animal models and comparable to what has been reported in humanized transgenic mouse lines. These features make the vole model a valuable and inexpensive tool for in vivo bioassays of the most common forms of sCJD and gCJD and for the characterization of human prion strains in relation to the clinico-pathological phenotype of patients. On this point, we infer the existence of at least two different prion strains in MM1/MV1 and MM2 sCJD, based on the survival times and the disease phenotype observed after primary and secondary transmission. The lack of transmission up to 500 dpi for MV2 and VV2 sCJD sources suggests that they could represent further sCJD strains. Genetic CJD with V210I and E200K mutations produced disease phenotypes similar to each other and indistinguishable from that induced by type 1 sCJD, and thus may be caused by a strain similar to the MM1/MV1 subtype. Overall, biological strain typing in voles concurs with the clinico-pathological classification of sCJD and gCJD [25,38].

The transmission studies presented here recapitulate several features of prion interspecies transmission, such as variable transmission barriers with different strains from the same donor species [11,15] and the occurrence of prion infections in clinically healthy animals [31,32]. The susceptibility of voles to different prion sources allowed us to study several transmission barriers in a single animal model. Surprisingly, V210I gCJD and E200K gCJD transmit in secondary transmission with mean survival times similar to the first passage. These findings show that some prion strains, but not all, could transmit to a different species (i.e., with a different PrP sequence) in the absence of a species barrier. Scott and colleagues recently proposed that the majority of the so-called species barriers are actually strain barriers [39].

Our results provide further evidence that the outcome of interspecies transmission of prions cannot be predicted by the degree of PrP sequence homology between two species, but depends mainly on the prion strain. In fact, prion strains coming from the same species display variable transmission barriers in voles (Figure 5C) as reported in mice [15] and transgenic models [16,20], consistent with the view that sequence homology alone is not sufficient to explain the species-barrier effect. Furthermore, transmission barriers in voles seem to vary according to the prion strain, and also when a given strain is propagated in different species (Figure 5D). Several studies with mammalian and yeast prions point to an intimate relationship between prion strains and transmission barriers [16,40–42]. This interpretation is consistent with recent studies with mammalian prion amyloids [43] and yeast prions [44], which suggest that the PrP sequence of any individual species dictates the range of possible PrP conformations and hence the susceptibility to different prion strains. In this context, it is possible to speculate that the vole PrP sequence is particularly prone to faithfully reproduce the conformation that characterizes PrP^Sc^ of some human CJD strains, as supported by the fact that gCJD cases do not encounter apparent transmission barriers. It is, however, possible that mechanisms other than the PrP primary sequence contribute to the species barrier, for instance binding of PrP^C^ [45] or PrP^Sc^ [46,47] to other proteins or to cellular factors. Transgenic mice expressing vole PrP are being generated and will be challenged with human prions in order to investigate whether the transmission barrier encountered in voles depends on vole PrP sequence or on other host factors.

Our findings also have important implications for public-health issues related to the zoonotic potential of prion diseases. The observation that prion infections in clinically healthy individuals of one species may be highly infectious for another species shows that a possible threat to human health can derive from animals in which a prion infection could not be easily detected by current diagnostic methods. Furthermore, the finding that prions can be transmitted between species with different PrP sequences, in the absence of a transmission barrier, underlines the difficulty of predicting the impact on public health of ruminant TSEs, such as BSE, BASE (bovine amyloidotic spongiform encephalopathy) [48], scrapie, and chronic wasting disease. The vole model, being susceptible to a range of prion strains, represents a major advance for the characterization of TSE isolates from different species and may aid in the development of control strategies for TSEs.

## Materials and Methods

### Animals.

The research protocol has been approved by the Service for Biotechnology and Animal Welfare of the Istituto Superiore di Sanità and authorized by the Italian Ministry of Health, according to Legislative Decree 116/92, which has implemented in Italy the European Directive 86/609/EEC on laboratory animal protection. Bank voles (Istituto Superiore di Sanità breeding colony) and C3H mice (Charles River, Como, Italy) were housed in standard cages and treated according to Legislative Decree 116/92 guidelines, and animal welfare was routinely checked by veterinarians from the Service for Biotechnology and Animal welfare. All animals were individually identified by a passive integrated transponder.

### Brain inocula.

Patient CJD diagnoses were confirmed by histopathology, immunohistochemistry, and Western blotting [27]. Human brain samples for transmission were collected from areas showing pathology and PrP^Sc^ accumulation, and stored at −80 °C.

For natural sheep scrapie isolates, frozen brain tissue from the medulla oblongata was obtained from one Sarda sheep (Italy) and one Suffolk sheep (United Kingdom) carrying the ARQ/ARQ (indicating amino acids at codons 136, 154, and 171, respectively, on both alleles) PrP genotype. Brain tissue from the United Kingdom scrapie case (PG671/97) was provided by the Veterinary Laboratory Agency (Weybridge, United Kingdom).

Brain tissue from a Cheviot sheep (AHQ/AHQ) experimentally infected with BSE was obtained from the Neuropathogenesis Unit at the Institute for Animal Health.

Mouse-passaged TSE strains were supplied by the TSE Resource Centre, Institute for Animal Health; hamster-passaged scrapie strains were originally donated by R. H. Kimberlin (Institute for Animal Health).

The inocula from mouse-adapted and hamster-adapted TSE strains were prepared from individual brains obtained from terminally ill C57BL mice (ME7, 139A, and 301C), VM mice (22A and 301V), and golden hamsters (139H and 263K).

For secondary passages in C3H mice and voles, the inocula were prepared from individual animals with terminal disease sacrificed after primary transmission, or from PrP^Sc^-positive mice sacrificed without signs of disease, as appropriate.

### Preparation of brain homogenates and bioassays.

New dedicated glassware and instruments were autoclaved at 136 °C for 1 h before use. All samples were homogenized at 10% (w/v) concentration in sterile physiological saline and stored at −80 °C. Groups of 10–20 bank voles or 5–15 C3H mice were injected by the intracerebral route (20 μl) into the left cerebral hemisphere under ketamine anesthesia.

Beginning 1 mo after inoculation, voles and mice were examined twice per week until the appearance of neurological signs, and were then examined daily. The animals were sacrificed with carbon dioxide when they reached the terminal stage of the disease. Survival time was calculated as the interval between inoculation and sacrifice.

### Histopathology, immunohistochemistry, and PET blot.

At post-mortem, each brain was divided into two parts by a sagittal paramedian cut. The smaller portion was immediately frozen and stored at −20 °C for Western blotting. The remaining part was immersed and fixed in 10% formol saline for 4 d. The brains were trimmed at standard coronal levels, decontaminated with formic acid for 1 h, and embedded in paraffin. Sections (6 μm thick each) were cut for haematoxylin and eosin staining, immunohistochemistry, and PET blot, randomly mixed and coded for pathological assessment.

For the construction of lesion profiles, vacuolar changes were scored in nine grey-matter areas of the brain on haematoxylin and eosin-stained sections, as described by H. Fraser and A. G. Dickinson [49]. Vacuolation scores are derived from at least six individual voles per group and from three individual mice per group, and are reported as means ± standard error of the mean.

For PrP immunohistochemistry, sections were collected on silanized slides (Dako-Cytomation, Glostrup, Denmark). After treatment at 60 °C for 24 h, sections were hydrated, pretreated with 98% formic acid for 1 min, followed by hydrating autoclaving for 30 min at 121 °C, and finally cooling overnight. Incubation with antibodies, plus avidin–biotin complex treatment and revelation, were carried out with Dako-Autostainer (Dako-Cytomation). Sections were treated with 6% normal goat serum (Vector, Burlingame, California, United States) in PBS for 30 min. Immunohistochemical detection of PrP was performed with mAb SAF84 (Spi-Bio, Montigny Le Bretonneux, France) at 2 μg/ml in PBS with 3% of normal goat serum (Vector) for 45 min. After washing with PBS, sections were treated with ABC Complex (Vector) for 45 min and with diaminobenzidine (Dako-Cytomation) for 7 min. Sections were counterstained with Mayer's haematoxylin. In each run, positive- and negative-control sections were included.

Sections for PET blot were collected on prewetted 0.45-μm-pore nitrocellulose membranes (Schleicher & Schuell, Dassel, Germany). Membranes were dried for 24 h at 55 °C. Membrane treatments, proteinase K digestion (50 μg/ml), and immunodetection were performed as described [50]. Monoclonal Ab SAF84 (1 μg/ml) was used as primary antibody.

### Western blot.

Brain tissues were homogenized (10% w/v) in 100 mM Tris-HCl (pH 7.4) containing 2% Sarcosyl (Sigma, St. Louis, Missouri, United States). The homogenates were incubated for 30 min at 37 °C with gentle shaking; proteinase K (50 μg/ml; Sigma) was added, and then the homogenates were further incubated for 1 h at 37 °C with gentle shaking. Protease treatment was stopped with 3 mM PMSF (Sigma). Treated homogenates were denatured by adding an equal volume of 2× NuPage sample buffer (Invitrogen, Carlsbad, California, United States) and heating for 10 min at 90 °C. After centrifugation at 12,000 rpm for 5 min in a microfuge, 10 μl of each sample was loaded onto 12% bis-Tris polyacrylamide gels (Invitrogen). Precision Plus Strep-tagged molecular markers (Bio-Rad, Hercules, California, United States) were loaded in each gel. After electrophoresis and Western blotting on polyvinylidene difluoride membranes (Millipore, Bedford, Massachusetts, United States), the blots were blocked in PBS containing 0.05% Tween 20 and 0.5% non-fat milk powder for 1 h. PrP^Sc^ from voles and mice was detected with mAb SAF84 (0.4 μg/ml) for 1 h at room temperature. When human PrP^Sc^ was analyzed together with vole PrP^Sc^, mAb SAF84 was used in combination with mAb 3F4 (1:5,000). Horseradish peroxidase-conjugated anti-mouse immunoglobulin (Pierce Biotechnology, Rockford, Illinois, United States) was used as secondary antibody (1:80,000 for 1 h). The membranes were developed with an enhanced chemiluminescence method (SuperSignal Femto, Pierce Biotechnology) and detected with the VersaDoc imaging system (Bio-Rad). Apparent molecular weights and glycoform patterns were determined with QuantityOne software (Bio-Rad). For glycoform analysis, values are derived from PrP^Sc^ of human inocula and from five individual voles per group (means ± standard error of the mean).

Samples that were negative by this standard protocol were further analyzed after PrP^Sc^ concentration. In this case, 10% (w/v) homogenates were added with 100 mM Tris-HCl (pH 7.4) containing Sarcosyl (Sigma) to obtain a final 5% (w/v) homogenate with 10% Sarcosyl. The homogenates were incubated for 20 min at room temperature and then centrifuged at 22,000 *g* for 20 min (TLA 100.3 rotor, Beckman Instruments, Fullerton, California, United States). Supernatants were added with proteinase K (50 μg/ml; Sigma) and incubated for 1 h at 37 °C with gentle shaking. Protease treatment was stopped with 3 mM PMSF (Sigma). The treated homogenates were ultracentrifuged at 210,000 *g* for 40 min (TLA 100.3 rotor, Beckman Instruments), and the pellets were resuspended in 100 μl of distilled water and desiccated in speed-vacuum (Speed Vac Sc 110, Savant, Holbrook, New York, United States) overnight. The final pellets were resuspended in NuPage sample buffer and were analyzed by Western blotting as above.

## Supporting Information

Figure S1 Neurodegeneration and PrP^Sc^ Deposition in Voles Infected with MM2 sCJD and MM1 sCJD (1.7 MB PDF)

### Accession Numbers

The GenBank (http://www.ncbi.nlm.nih.gov/Genbank) accession numbers for the genes and gene products discussed in this paper are C57Bl mice PrP (M18070), hamsters PrP (M14054), human PrP (M13899), VM mice PrP (M18071), vole PrP (AF367624), and sheep PrP (M31313).

## Abbreviations

BASE: bovine amyloidotic spongiform encephalopathy
BSE: bovine spongiform encephalopathy
CJD: Creutzfeldt–Jakob disease
dpi: days post-infection
gCJD: genetic Creutzfeldt–Jakob disease
GSS: Gerstmann–Sträussler–Scheinker disease
M: methionine
PET: paraffin-embedded tissue
sCJD: sporadic Creutzfeldt–Jakob disease
TSE: transmissible spongiform encephalopathy
V: valine
vCJD: variant Creutzfeldt–Jakob disease
