# Supplementary figures and images for: Efficient Transmission and Characterization of Creutzfeldt–Jakob Disease Strains in Bank Voles
Source: PLoS Pathog. 2006 Feb 24;2(2):e12. doi: 10.1371/journal.ppat.0020012 (PMC1383487; doi:10.1371/journal.ppat.0020012)

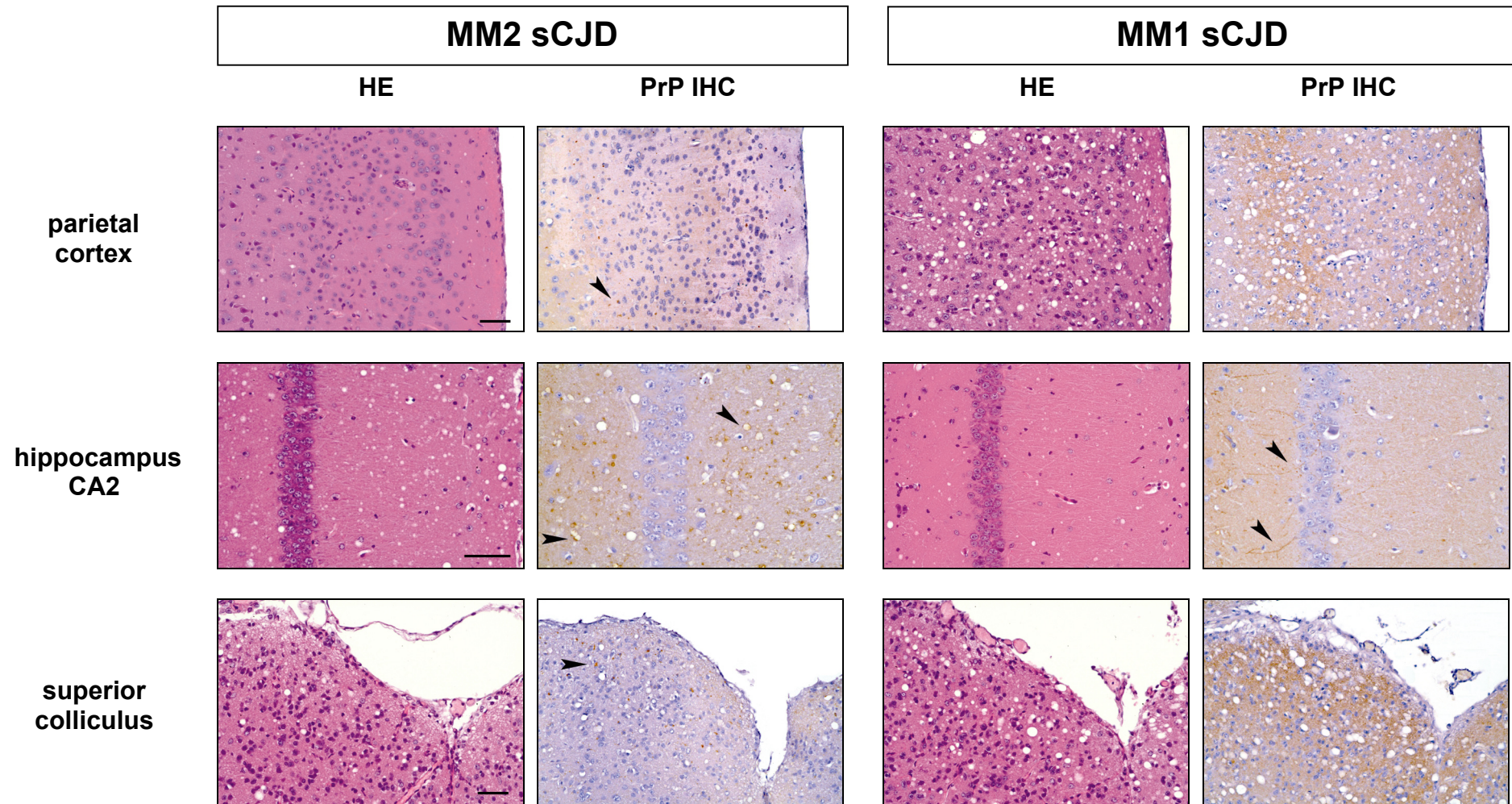

Supplement: Figure S1 — (1.7 MB PDF) [file ppat.0020012.sg001.pdf]
